# Supplementary material for: Sequential and Binomial Sampling Plans to Estimate Thrips tabaci Population Density on Onion
Source: Insects. 2021 Apr 8;12(4):331. doi: 10.3390/insects12040331 (PMC8068047; doi:10.3390/insects12040331)
Supplement: Supplementary file 1 [file insects-12-00331-s001.pdf]

Sequential and binomial sampling plans to estimate *Thrips tabaci* population density on onion

Supplementary material: List of sampled sites including onion variety, plant phenology, date and sampling season.

Plot	State	Latitude	Longitude	Date	Sampling Season	Onion Variety	Plant Phenology (BBCH code)
1	Michoacán	19°52'59.42"N	101°12'15.84"W	07/02/2015	Winter	Carta Blanca	LD (16)
2	Michoacán	19°54'10.43"N	101°14'16.29"W	07/02/2015	Winter	Carta Blanca	LD (17)
3	Michoacán	19°57'19.29"N	100°27'24.82"W	13/02/2015	Winter	Carta Blanca	LD (17)
4	Michoacán	19°57'48.52"N	100°27'40.67"W	13/03/2015	Winter	Carta Blanca	LD (16)
5	Michoacán	19°58'4.96"N	100°27'28.12"W	14/03/2015	Winter	Carta Blanca	LD (17)
6	Estado de México	18°47'13.29"N	99°37'24.69"W	03/10/2015	Fall	Sterling	DHVPP (43)
7	Estado de México	18°46'24.69"N	99°37'23.93"W	03/10/2015	Fall	Sterling	DHVPP (43)
8	Estado de México	18°47'7.23"N	99°37'3.69"W	10/10/2015	Fall	Sterling	DHVPP (43)
9	Michoacán	19°52'59.42"N	101°12'15.84"W	20/02/2016	Winter	Carta Blanca	LD (18)
10	Michoacán	19°53'23.90"N	101°12'12.25"W	20/02/2016	Winter	Carta Blanca	LD (17)
11	Michoacán	19°54'10.43"N	101°14'16.29"W	21/02/2016	Winter	Carta Blanca	LD (17)
12	Michoacán	19°53'50.61"N	101°13'59.97"W	21/02/2016	Winter	Carta Blanca	LD (16)
13	Michoacán	19°57'19.29"N	100°27'24.82"W	27/02/2016	Winter	Carta Blanca	LD (17)
14	Michoacán	19°57'48.52"N	100°27'40.67"W	27/02/2016	Winter	Carta Blanca	LD (16)
15	Michoacán	19°58'4.96"N	100°27'28.12"W	04/03/2016	Winter	Carta Blanca	LD (18)
16	Puebla	18°59'1.33"N	97°52'27.65"W	12/03/2016	Winter	Carta Blanca	LD (16)
17	Puebla	18°59'55.71"N	97°50'22.91"W	13/03/2016	Winter	Carta Blanca	LD (17)
18	Puebla	19°00'53.98"N	97°51'50.17"W	19/03/2016	Winter	Carta Blanca	LD (19)
19	Estado de México	18°47'7.23"N	99°37'3.69"W	17/09/2016	Fall	Sterling	DHVPP (43)
20	Estado de México	18°47'13.29"N	99°37'24.69"W	24/09/2016	Fall	Sterling	DHVPP (43)
21	Estado de México	18°46'24.69"N	99°37'23.93"W	24/09/2016	Fall	Sterling	DHVPP (43)
22	Michoacán	19°52'59.42"N	101°12'15.84"W	17/02/2017	Winter	Carta Blanca	LD (16)
23	Michoacán	19°54'10.43"N	101°14'16.29"W	17/02/2017	Winter	Carta Blanca	LD (16)
24	Michoacán	19°53'23.90"N	101°12'12.25"W	18/02/2017	Winter	Carta Blanca	LD (16)
25	Puebla	18°59'1.33"N	97°52'27.65"W	18/03/2017	Winter	Carta Blanca	LD (16)
26	Puebla	18°59'55.71"N	97°50'22.91"W	18/03/2017	Winter	Carta Blanca	DHVPP (43)
27	Puebla	19°00'53.98"N	97°51'50.17"W	19/03/2017	Winter	Carta Blanca	DHVPP (43)
28	Puebla	18°57'58.42"N	97°52'58.19"W	19/03/2017	Winter	Carta Blanca	DHVPP (43)
29	Estado de México	18°47'13.29"N	99°37'24.69"W	14/10/2017	Fall	Sterling	DHVPP (43)
30	Estado de México	18°46'24.69"N	99°37'23.93"W	14/10/2017	Fall	Sterling	DHVPP (43)
31	Estado de México	18°47'7.23"N	99°37'3.69"W	19/10/2017	Fall	Sterling	DHVPP (43)
32	Michoacán	19°52'59.42"N	101°12'15.84"W	25/02/2018	Winter	Carta Blanca	LD (18)
33	Michoacán	19°53'23.90"N	101°12'12.25"W	25/02/2018	Winter	Carta Blanca	LD (18)

34	Michoacán	19°54'10.43"N	101°14'16.29"W	03/03/2018	Winter	Carta Blanca	LD (18)
35	Puebla	19°00'53.98"N	97°51'50.17"W	04/03/2018	Winter	Carta Blanca	LD (18)
36	Puebla	18°59'1.33"N	97°52'27.65"W	05/03/2018	Winter	Carta Blanca	LD (17)
37	Puebla	18°59'55.71"N	97°50'22.91"W	05/03/2018	Winter	Carta Blanca	LD (17)
38	Puebla	18°57'58.42"N	97°52'58.19"W	17/03/2018	Winter	Carta Blanca	DHVPP (43)
39	Estado de México	18°47'13.29"N	99°37'24.69"W	20/10/2018	Fall	Sterling	DHVPP (43)
40	Estado de México	18°46'24.69"N	99°37'23.93"W	20/10/2018	Fall	Sterling	DHVPP (43)
41	Estado de México	18°47'7.23"N	99°37'3.69"W	21/10/2018	Fall	Sterling	DHVPP (43)
42	Michoacán	19°53'23.90"N	101°12'12.25"W	02/03/2019	Winter	Carta Blanca	LD (16)
43	Puebla	18°59'1.33"N	97°52'27.65"W	16/03/2019	Winter	Carta Blanca	DHVPP (43)
44	Estado de México	18°46'24.69"N	99°37'23.93"W	21/09/2019	Fall	Sterling	DHVPP (43)
45	Estado de México	18°47'7.23"N	99°37'3.69"W	27/09/2019	Fall	Sterling	DHVPP (43)
46	Michoacán	19°52'59.42"N	101°12'15.84"W	01/03/2020	Winter	Carta Blanca	LD (17)
47	Michoacán	19°53'23.90"N	101°12'12.25"W	01/03/2020	Winter	Carta Blanca	LD (19)
48	Michoacán	19°57'19.29"N	100°27'24.82"W	08/03/2020	Winter	Carta Blanca	DHVPP (43)
49	Puebla	18°59'1.33"N	97°52'27.65"W	14/03/2020	Winter	Carta Blanca	DHVPP (43)
50	Puebla	18°59'55.71"N	97°50'22.91"W	14/03/2020	Winter	Carta Blanca	DHVPP (43)
51	Estado de México	18°46'24.69"N	99°37'23.93"W	20/03/2020	Winter	Sterling	LD (16)

DHVPP: Development of harvestable vegetative plant parts

LD: Leaf development, main shoot
